# The Ochsner Way

**DOI:** 10.31486/toj.22.5026

**Published:** 2022

**Authors:** Ronald G. Amedee

**Affiliations:** Director of Clinical School and Professor, The University of Queensland Medical School, Ochsner Clinical School, New Orleans, LA; Editor-in-Chief, *Ochsner Journal*

*Dr Ochsner is credited with teaching more than 3,000 medical students and at least 200 surgeons, including the famous heart surgeon Michael DeBakey.*

*–Harold M. Schmeck, Jr.*

*(from the obituary for Dr Alton Ochsner in the* New York Times, *September 25, 1981)*

The Summer 2022 issue of the *Ochsner Journal* contains 6 original research articles, 2 quarterly columns, and 7 case reports/clinical observations that serve as the principal elements for this edition. In addition, please consider reading the letter to the editor submitted by a faculty leader in the Ochsner Clinical School Character in Medicine course on the subject of “Patient Care and Reciprocal Healing.”

Regarding original research, “Effectiveness of a Patient-Centered Dietary Educational Intervention” is presented by Ardoin, Hamer, Mason, et al, followed by “Rates of Cognitive Decline in 100 Patients with Alzheimer Disease” by Miyakawa-Liu, Feehan, Pai, and Garcia-Diaz. Urgent attention to mental health issues continues to predominate in our world, which is why “Impact of a Mental Health Diagnosis on Emergency Department Adherence to Sepsis Care Guidelines” by Crapanzano, Hammarlund, Musso, et al is such an important read. This paper is followed by a contribution from the surgical arena on “Hospital-Based Same-Day Compared to Overnight-Stay Mastectomy: An American College of Surgeons National Surgical Quality Improvement Program Analysis” by Sibia, Klune, Turcotte, et al. Oğlak, Ölmez, and Tunç offer “Evaluation of Antepartum Factors for Predicting the Risk of Emergency Cesarean Delivery in Pregnancies Complicated With Placenta Previa.” Finally, in the original research section, consider “Effect of Medicaid Expansion on Visit Composition in a Louisiana Health Care System” by Hamer, Mandala, Jones, et al.

Our quarterly columns include one from orthopedic sports medicine by DeStefano, Oberle, Donohoe, et al entitled “Optimizing Pain Control and Function in Patients With Adhesive Capsulitis by Choosing the Best Injection Site,” while the other comes from our radiology colleagues at Ochsner on “Autoimmune Encephalitis Presenting as Dystonia-Parkinsonism” authored by Milburn, Van Hook, and Steven.

Case reports contained in this issue include two manifestations of mucormycotic infection: “Lower Extremity Salvage in a Diabetic Patient With Cutaneous Mucormycosis and COVID-19 After Open Patella Fracture” by Hammoudi, Morar, Garbuzov, et al and “Bilateral Cerebral Mucormycosis in an Immunocompetent Female” submitted by Okwechime, Reyes, Trivedi, et al. Rounding out the case reports is “Dural-Based Posterior Fossa Medulloblastoma Mimicking a Petrous Meningioma in Late Adulthood” by Griepp, Miller, Klein, et al that highlights the manifestations of this unusual clinical scenario.

This year we are celebrating the 80^th^ anniversary year of Ochsner's founding. When I read Dr Alton Ochsner's obituary recently, I was struck by the number of patient lives (he performed 20,000 operations during his illustrious career) and learner lives he so critically impacted during the time he walked the halls of his namesake institution. Dr Ochsner and his 4 cofounders were innovative medical pioneers who envisioned and practiced in an evolving medical culture. The tools and knowledge we have today make us far more capable of diagnosing and treating complicated ailments than the founders had available to them in the 1940s and 1950s. Nevertheless, they brought vision, planning, and diligent execution to their care for patients, and the patients we are privileged to care for at Ochsner Health in 2022 are the beneficiaries of their extraordinary legacy. That legacy is why today it is so critically important that we collectively strive to continue to train the next generation of clinicians in “The Ochsner Way.”

